# Knowledge, Attitudes, and Practices Toward Deaf Patients Among Healthcare Workers in Saudi Arabia: 2020-2021

**DOI:** 10.7759/cureus.49655

**Published:** 2023-11-29

**Authors:** Nurah Alamro, Shahad Alsahil, Renad Alhaqbani, Nouf Alburaykan, Najoud Alali

**Affiliations:** 1 Family and Community Medicine, King Saud University Medical City, Riyadh, SAU; 2 College of Medicine, King Saud University, Riyadh, SAU; 3 Medicine, King Saud University Medical City, Riyadh, SAU

**Keywords:** practice, knowledge, healthcare worker, deaf, attitude

## Abstract

Introduction

The literature informs us that people who are Deaf frequently struggle with health services, which contributes to lower health literacy due to communication and language barriers. Few health professionals understand sign language, so communication between a Deaf person and a health professional may rely on a mediator, usually a family member or an interpreter, to provide communication assistance.

Aim

This study aimed to assess the knowledge, attitudes, and practices (KAP) of healthcare workers (HCWs) at King Saud University Medical City (KSUMC) toward Deaf adult patients.

Subjects and methods

This cross-sectional study was conducted among HCWs at KSUMC, Saudi Arabia. A self-administered questionnaire was sent to the HCWs using an online survey. The questionnaire includes socio-demographic data (i.e., age, gender, marital status, etc.), previous interaction with Deaf patients, and KAP items.

Results

Of the 351 HCWs, 63.8% were females, and 41.6% were aged between 28 and 37 years old. The overall mean knowledge score was 1.14 out of 7 points. The overall mean attitude score was 46.2 out of 90 points, and the mean practice score was 19.1 out of 35 points. Significant factors of increased attitude include being a non-Saudi, being married, having children, increasing years of working experience, and being a nurse, while being a male, having previous interaction with a Deaf patient, and having skills in any type of sign language were the significant predictors of increased practice. Working in the surgery department was the only predictor associated with increased knowledge.

Conclusion

There was a significant deficiency, particularly with regard to knowledge and attitude toward Deaf patients. Increasing knowledge was associated with increasing practices but not with attitude. HCWs who had previous interactions with Deaf patients and had knowledge of any sign language tended to demonstrate better practice in dealing with Deaf patients. Further, longitudinal studies are needed to determine the level of KAP among HCWs in our region.

## Introduction

Many factors affect access to health information and healthcare services. One of these factors is communication quality [1]. The quality of health services is affected by the attitudes of healthcare workers (HCWs) toward persons with disabilities [2]. If they have a misunderstanding about disability or have inadequate experience and knowledge about managing a disability, this could lower the quality of services provided to persons with disabilities [3].

Communication issues are almost everywhere, but these issues become more significant when involving language and cultural barriers [4]. Hearing loss is one of the most important disabilities that affect health status and communication ability, making it challenging to understand basic conversations [5]. Furthermore, communication obstacles restrict a deaf person's health experience.

Deaf and hard of hearing are defined as an "inability to hear normally", which is a general term that includes the term Deaf with capital D that refers to those "who identify with and participate in the culture, society, and language of Deaf people, which is based on Sign Language" [6].

Hearing loss is a pervasive issue that significantly influences healthcare experiences across diverse populations. The prevalence of hearing impairment has escalated in recent years. According to WHO, the percentage of deaf and hard-of-hearing people worldwide is over 5%, which equals around 466 million people in the world [7]. By 2050, as rough calculations reveal, one in every 10 people will have disabling hearing loss [7]. Few studies have investigated the prevalence of hearing impairment in Middle Eastern countries. In Saudi Arabia, as indicated by the General Authority for Statistics, the prevalence of disabling hearing loss among the Saudi population is 1.4% [8]; this is a realistic indicator concerning the spread of disabling hearing loss. Of these deaf people in the Kingdom, sign language (SL) users who are five years old and above account for 27,748 [8]. According to the Ministry of Health, deaf people in the Kingdom are hugely neglected, especially in public departments and places, because of the absence of interpreters [9].

Individuals with hearing loss often encounter barriers in understanding medical information, leading to potential misunderstandings about diagnoses, treatment plans, and post-care instructions. The literature informs us that people who are deaf frequently struggle with health services, which contributes to lower health literacy due to communication and language barriers [2]. For instance, a study shows that 76% of surveyed deaf adults did not know the normal body temperature. Also, 41% of the original group cannot understand simple medical prescriptions [10]. If patients are unable to sufficiently convey the history of their condition, there is a possibility of failure to make a correct diagnosis and prescribe appropriate treatment [1]. The resulting inequalities make the health outcomes of deaf patients likely to be worse than hearing people with the same condition [11].

Few health professionals understand SL, and therefore, communication between a Deaf person and a health professional may rely on a mediator, usually a family member or an interpreter, to provide communication assistance [1]. Although interpreters help overcome language barriers, inappropriate use of escorts as interpreters may steal patients' independence and confidentiality rights [4]. In the absence of communication assistance, HCWs usually communicate in writing [12], despite knowing the disadvantages of this method, such as patient literacy-level dependency and time consumption [13]. According to one study, only 20% of deaf people have fluency in written English [14]. Nevertheless, there is no one best accommodation for all deaf and hard-of-hearing people [15].

Indicators of healthcare quality don't usually consider deafness or the use of SL; therefore, the full impact of healthcare access inequality on the health of deaf SL users and their families is almost unknown [11]. Consequently, access to healthcare affects the health of deaf people, and a call for action to provide better access to health services is essential [16]. The current healthcare system still has a long way to go to improve the health services provided for deaf patients [15]. Fortunately, these improvements are achievable; it just takes meaningful action. Some studies indicate that the exposure of HCWs to Deaf patients may positively impact cultural competency [17] and attitudes toward Deaf patients [18,19].

The knowledge, attitudes, and practices (KAP) of HCWs may have a notable impact on all aspects of care and the use of services by deaf adults. There are limited studies that have led to an understanding of HCWs' KAP toward deaf adult patients in the Middle East, including Saudi Arabia. Consequently, this study aims to assess the KAP of HCWs toward deaf patients as well as the association between the level of KAP with selected demographic factors. Understanding this would enable policymakers and HCWs to develop interventional approaches to drive more attention toward deaf people, therefore enhancing their healthcare experience.

## Materials and methods

The study was designed as a cross-sectional population-based anonymous electronic survey and conducted during the period from October 2020 to February 2021. Data from the present study were collected using a convenience sampling technique with a target sample consisting of 440 HCWs in different specialties at King Khalid University Hospital and King Abdul-Aziz University Hospital in Riyadh, Saudi Arabia. The sample size calculation was based on a chi-squared test with an effect size of 0.1, and a power of 80% will require a total sample of 362 at 5% levels using a two-tailed test. A 20% increase in the sample size was added to account for non-response and incomplete responses, making a total sample size of 434, which we will round to a sample size of 440. Of the 440 HCWs approached, 350 took part in the study (80%). This study has been reviewed and received ethics clearance from King Saud University Medical City on October 11, 2020 (approval number: E-20-5215). Participation in the study was voluntary and anonymous.

This study used an English questionnaire found in the Appendices section consisting of five sections: consent, demographics, knowledge, attitude, and practice. All these sections were taken from previous questionnaires that had been used before and adapted to be Saudi Arabia-focused [12,19-21]. The first section explained the purpose of the study in English at the beginning of the electronic survey. The respondent is allowed to ask questions via a dedicated email address for any inquiries regarding the study. Electronic consent was requested since the study presents no more than minimal risk of harm to subjects and involves no procedures for which written consent is normally required outside the study context. Personal identifiers are not collected, which ensures confidentiality. The second section was designed to collect demographic data about the participants. The knowledge section was obtained from a study questionnaire [19] and modified to apply to all HCWs. The attitude section was derived from three different studies [19-21] and measured by the Scale of Attitudes toward Disabled Persons (SADP). Participants are required to respond on a 6-point Likert scale indicating to what extent they agree or disagree with statements. The practice section was adapted from two studies [12,21]. It contains eight items with 5-point Likert scales from always to never. The last question accesses the most common method used during communication with a deaf patient. The questionnaire submitted with this form was piloted using 20 participants who are HCWs from Saudi Arabia for its legibility and comprehensibility. The anonymous electronic survey was distributed to HCWs through emails and social media.

Data collection was completed using electronic surveys utilizing the English questionnaire. Data from questionnaires was transferred into an Excel database. All data were cleaned and analyzed using IBM SPSS Statistics for Windows, Version 24.0 (Released 2016; IBM Corp., Armonk, New York, United States). Descriptive statistics are used to describe the quantitative and categorical variables. Pearson's chi-squared test was used to assess the association between the categorical variables. Both Student t-tests and one-way analysis of variance were used for comparing means. Linear regression analysis was used. A p-value of 0.05 and 95% confidence intervals were used to report the statistical significance and precision of the results.

The potential risk of a loss of confidentiality was minimized by the use of survey identification numbers and the anonymous questionnaire with no personal identifiers at the time of data entry. There are no anticipated risks or discomforts. Patients were not involved in any part of our research's design, conduct, reporting, or dissemination plans. However, HCWs were involved in the research by answering the survey questionnaire.

Questionnaire criteria

Knowledge about deafness was assessed using a seven-item questionnaire, with the correct answer identified and coded with 1, while the incorrect answer was coded with 0. The overall scores of knowledge have been calculated by adding all seven items. A possible score range from 0 to 7 points has been generated. The greater the score, the greater the knowledge about deafness. By using 50% and 75% as cutoff points to determine the level of knowledge, parents were categorized as having poor knowledge if the score was below 50%, 50-75% were categorized as moderate, and above 75% were categorized as good knowledge levels.

The attitude toward deafness has been assessed by using an 18-item questionnaire, with 5-point Likert scale categories ranging from "strongly disagreed," coded with 1, to "strongly agreed," coded with 5, as the answer options. Negative questions have been re-coded reversely to avoid bias in the score. The total attitude has been calculated by adding all 18 items. The higher the score, the higher the attitude toward deafness. By applying similar criteria of 50% and 75% as cutoff points, parents were considered negative if the score was below 50%, 50-75% were considered neutral, and above 75% were considered positive attitude level.

Lastly, the practice of deafness has been assessed using a seven-item questionnaire, with 5-point Likert scale categories ranging from "never" coded with 1 to "always" coded with 5 as the answer options. Similar criteria taken from the knowledge were applied. The practice score has a score range from 7 to 35 points. 

Statistical analysis

Descriptive statistics were computed and reported as frequencies and proportions (%) for qualitative variables and mean±standard deviation (SD) for quantitative variables. The differences in the KAP scores in regard to the socio-demographic characteristics and previous interaction with Deaf patients have been performed using the Mann-Whitney Z-test and Kruskal-Wallis H-test. Statistical collinearity was measured using the Shapiro-Wilk test and the Kolmogorov-Smirnov test. The KAP scores follow the non-normal distribution. Thus, the non-parametric tests were applied. The Spearman correlation coefficient was also used to determine the correlation between the KAP scores. Statistical significance was set at p<0.05. The data analyses were performed using IBM SPSS Statistics for Windows, Version 26.0 (Released 2019; IBM Corp., Armonk, New York, United States).

## Results

Socio-demographic characteristics of HCWs 

This survey recruited 351 HCWs. As described in Table 1, 41.6% were aged between 28 and 37 years old, with females being dominant (63.8%). Nearly two-thirds (62.1%) were Saudis, and 55.3% were single. HCWs who have children constitute 36.2%. Among those with children (n=127), four of them had a disability, with developmental delay, autism spectrum disorder (ASD), and autism detected in three children. HCWs who had one to five years of working experience constitute 29.6%. The most common specialty was nurse (22.5%), followed by physician (27.4%) and intern (15.1%).

**Table 1:** Socio-demographic characteristics of HCWs (n=351) HCWs: healthcare workers; ASD: autism spectrum disorder

Study data	N (%)
Age group	
18-27 years	134 (38.2%)
28-37 years	146 (41.6%)
38-47 years	52 (14.8%)
48-57 years	16 (04.6%)
>57 years	3 (0.90%)
Gender	
Female	224 (63.8%)
Male	127 (36.2%)
Nationality	
Saudi	218 (62.1%)
Non-Saudi	133 (37.9%)
Marital status	
Single	194 (55.3%)
Married	151 (43.0%)
Divorced	6 (01.7%)
Having children	
Yes	127 (36.2%)
No	224 (63.8%)
Does any of your children have any disability? (n=127)	
Yes	4 (03.1%)
No	123 (96.9%)
Type of children's disability (n=4)	
Developmental delay due to necrotizing encephalitis	1 (25.0%)
ASD	1 (25.0%)
Autism	1 (25.0%)
Not mentioned	1 (25.0%)
Years of working experience	
<1 year	95 (27.1%)
1-5 years	104 (29.6%)
6-10 years	71 (20.2%)
>10 years	81 (23.1%)
Specialty	
Nurse	118 (33.6%)
Physician	96 (27.4%)
Intern	53 (15.1%)
Nutritionist	12 (03.4%)
Pharmacist	11 (03.1%)
Technician	10 (02.8%)
Dentist	7 (02.0%)
Laboratory worker	6 (01.7%)
Physiotherapist	3 (0.90%)
Other	35 (10.0%)

**Figure 1:**
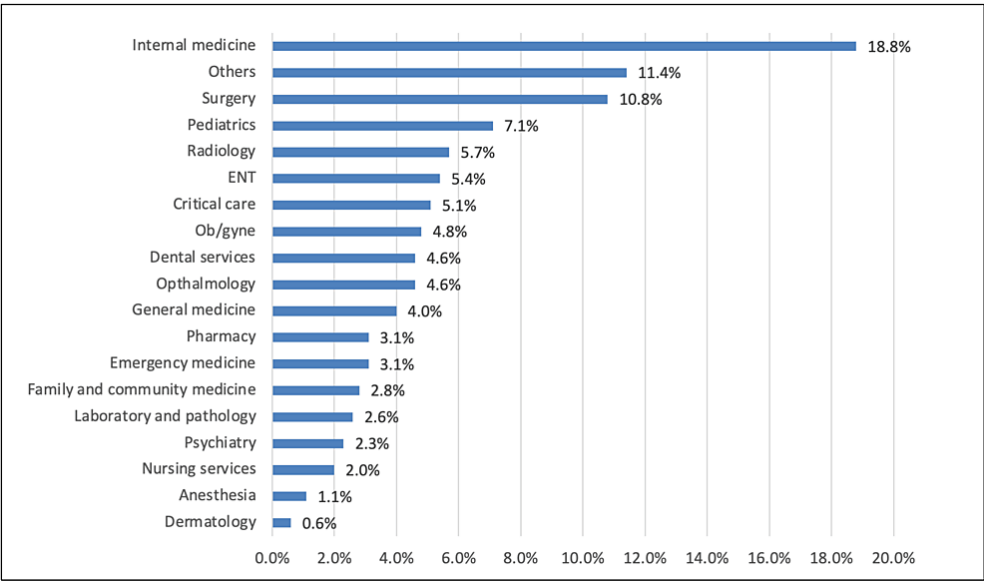
HCWs' department HCWs: healthcare workers

In Figure 1, the most common department was internal medicine (18.8%), followed by the surgery department (10.8%) and pediatrics (7.1%), while dermatology was the least (0.6%).

In Table 2, 10.5% of the HCWs indicated regular interaction with someone who is Deaf, with family members being the most common (35.1%). The prevalence of HCWs who had previous interaction with Deaf patients was 53.3%, while the prevalence of those who knew any SL was 15.4%. Among them, 48.1% knew SL by hand, which they mostly learned from online/self-study (48.1%).

**Table 2:** Interaction with Deaf patients and sources of information on sign language skills (n=351)

Variables	N (%)
Do you have any interaction on a regular basis with someone who is deaf besides the working environment?	
Yes	37 (10.5%)
No	314 (89.5%)
Please specify the interaction with whom (n=37)	
Family member	13 (35.1%)
Community	11 (29.7%)
Patients	9 (24.3%)
Friend	2 (05.4%)
Workplace	2 (05.4%)
Have you had any previous interactions with a Deaf patient?	
Yes	187 (53.3%)
No	164 (46.7%)
Do you have any type of sign language skills?	
Yes	54 (15.4%)
No	297 (84.6%)
What type of sign language do you possess? (n=54)	
Sign language by hand	26 (48.1%)
Sign language by facial expression and body language	3 (05.6%)
Writing and hand movement	2 (03.7%)
Others	5 (09.3%)
Not mentioned	18 (33.3%)
From where did you learn your sign language skills? (n=54)	
Family and friends	20 (37.0%)
College/community course	8 (14.8%)
Online/self-study	26 (48.1%)

KAP toward deafness

Regarding knowledge items (Table 3), a lack of knowledge among our HCWs is evidently seen in our results, most notably, the prevalence of signing Deaf people in Saudi Arabia (correct answer: 4.6%) and the prevalence of deafness in Saudi Arabia (correct answer: 3.1%). Surprisingly, none of the HCWs knew about the common causes of deafness and the percentage of Deaf children who have hearing parents. The overall mean knowledge score was 1.14 (SD: 1.02), poor and moderate, consisting of 88.6% and 11.4%, respectively. None of the HCWs were considered to have a good knowledge level. Regarding attitude, the top three statements with the highest ratings were "Deaf people should receive help in their home environment" (mean score: 3.96), followed by "Communicating with Deaf people makes me feel uncomfortable" (mean score: 3.66) and "Communicating with Deaf people would make me very nervous" (mean score: 3.52). The overall mean attitude score was 46.2 (SD: 6.09). Accordingly, negative and neutral attitudes were found in 36.8% and 63.2%, respectively. None of the HCWs were found to have positive attitude levels. Finally, regarding practice, the top three statements with the highest ratings were "I am able to effectively get Deaf patients' attention" (mean score: 3.38), "I am able to refrain from "offensive" actions or behaviors such as exaggerating speech, interrupting, and talking over a Deaf patient" (mean score: 2.95) and "I am able to introduce myself to Deaf patients" (mean score: 2.39). The total mean practice score was 19.1 (SD: 6.42), with poor, moderate, and good practice levels detected in 40.2%, 45.9%, and 14%, respectively.

**Table 3:** Assessment of knowledge, attitude, and practice toward deafness (n=351) †: reversed coded question; SD: standard deviation Attitude items have a category range from "strongly disagree" coded with 1 to "strongly agree" coded with 5. Negative questions have been re-coded reversely to avoid bias in the score. Practice items have a category range from "never" coded with 1 to "always" coded with 5

Knowledge items	N (%)
What is the language most used by people who are deaf in Saudi Arabia? (Saudi Sign Language)	134 (38.2%)
What is the approximate prevalence of deafness in Saudi Arabia? (1%)	11 (3.1%)
Approximately how many signing Deaf people are there in Saudi Arabia? (30,000)	16 (4.6%)
Which of the following is a common cause of deafness? (All of the above)	0
Which of the following are Deaf people NOT allowed to do in Saudi Arabia? (Join the police force)	107 (30.5%)
What is the unit that measures deafness? (Decibel)	133 (37.9%)
What is the percentage of Deaf children who have hearing parents? (90%)	0
Total knowledge score (mean±SD)	1.14±1.02
Level of knowledge	
Poor	311 (88.6%)
Moderate	40 (11.4%)
Good	0
Attitude items	Mean±SD
Deaf people should receive help in their home environment	3.96±0.93
Communicating with Deaf people makes me feel uncomfortable^†^	3.66±0.99
Communicating with Deaf people would make me very nervous^†^	3.52±1.05
Deaf people should learn to lipread	3.37±0.87
I think communicating with Deaf people would be very hard for me^†^	3.26±0.97
I have no confidence in communicating with Deaf people^†^	2.87±1.11
Deaf people should learn speech rather than sign language	2.53±0.99
Deaf people should not be viewed as "impaired"	2.31±1.09
I think communicating with any Deaf would be enjoyable and stimulating	2.31±0.90
It is important to me to communicate with Deaf people	2.21±0.81
Knowing how to communicate with Deaf people will increase my opportunities	2.20±0.81
I am sure I could learn to communicate with Deaf people effectively	2.15±0.82
Once I started to communicate with Deaf people, I would probably want to have more opportunities to use it	2.12±0.76
I would like to take a sign language course to communicate with Deaf people	2.11±0.95
I would like to communicate with Deaf people	2.02±0.87
Learning about communicating with Deaf people is worthwhile	1.92±0.82
Interpreters should be available for Deaf people at hospitals	1.83±0.85
More research should be done to find cures for deafness	1.82±0.82
Total attitude score (mean±SD)	46.2±6.09
Level of attitude	N (%)
Negative	129 (36.8%)
Neutral	222 (63.2%)
Positive	0
Practice items	Mean±SD
I am able to effectively get Deaf patients' attention (tapped on shoulder, waved, etc.)	3.38±0.16
I am able to refrain from "offensive" actions or behaviors such as exaggerating speech, interrupting, and talking over a Deaf patient	2.95±1.37
I am able to introduce myself to Deaf patients	2.85±1.18
I am able to check for a Deaf patient's understanding	2.65±1.16
I am able to ask Deaf patients to wait for an interpreter	2.48±1.22
I am able to effectively gather the Deaf patients' complaints (feels sick, head hurts, etc.)	2.44±1.43
I am able to successfully recognize and understand the following Saudi Sign Language signs: pain, sick, and help	2.39±1.27
Total practice score (mean±SD)	19.1±6.42
Level of practice	N (%)
Poor	141 (40.2%)
Moderate	161 (45.9%)
Good	49 (14.0%)

When measuring the differences in the score of KAP in relation to the socio-demographic characteristics and previous interaction with Deaf patients (Table 4), it was revealed that a higher knowledge score was more associated with HCWs who were working in the surgery department (H=9.395; p=0.024). A higher attitude score was more associated with being a non-Saudi (Z=4.199; p<0.001), being married (Z=2.766; p=0.006), having children (Z=3.547; p<0.001), increasing years of working experience (Z=2.739; p=0.006), and being a nurse (H=20.681; p<0.001). Also, a higher practice score was more associated with being male (Z=2.066; p=0.039), having previous interaction with a Deaf patient (Z=3.440; p=0.001), and having skills in any type of SL (Z=2.299; p=0.022).

**Table 4:** Differences in the score of knowledge, attitude, and practice in relation to socio-demographic characteristics and previous interaction with Deaf patients (n=351) SD: standard deviation § P-value has been calculated using the Mann-Whitney Z-test ‡ P-value has been calculated using the Kruskal-Wallis H-test ** Significant at p<0.05 level

Factor	Knowledge score (7) mean±SD	Attitude score (90) mean±SD	Practice score (35) mean±SD
Age group			
18-27 years	1.12±1.09	45.3±6.37	19.0±6.46
28-37 years	1.12±0.98	46.6±5.84	19.1±6.63
>37 years	1.24±0.98	47.1±5.96	19.4±5.95
H-test; p-value^‡^	1.237; 0.539	5.493; 0.064	0.240; 0.887
Gender			
Female	1.11±0.99	46.2±5.94	18.7±6.54
Male	1.20±1.06	46.2±6.38	19.9±6.12
Z-test; p-value^§^	0.711; 0.477	0.045; 0.964	2.066; 0.039**
Nationality			
Saudi	1.11±1.08	45.1±6.17	19.4±6.59
Non-Saudi	1.19±0.92	47.9±5.59	18.6±6.09
Z-test; p-value^§^	1.312; 0.190	4.199; <0.001**	1.237; 0.216
Marital status			
Unmarried	1.17±1.09	45.3±6.34	19.2±6.30
Married	1.11±0.93	47.3±5.58	19.00±6.58
Z-test; p-value^§^	0.049; 0.961	2.766; 0.006**	0.223; 0.823
Having children			
Yes	1.13±0.93	47.8±5.37	19.2±6.22
No	1.15±1.07	45.2±6.29	19.1±6.54
Z-test; p-value^§^	0.167; 0.868	3.547; <0.001**	0.370; 0.711
Years of working experience			
≤5 years	1.09±1.06	45.3±6.41	18.9±6.49
>5 years	1.22±0.97	47.3±5.48	19.5±6.32
Z-test; p-value^§^	1.556; 0.120	2.739; 0.006**	1.061; 0.289
Specialty			
Nurse	1.17±0.92	47.9±5.50	19.0±6.73
Physician	1.19±1.09	44.1±5.75	19.5±5.65
Intern	1.38±1.23	45.6±6.87	18.9±6.71
Other allied specialties	0.88±0.88	46.5±6.11	19.0±6.69
H-test; p-value^‡^	7.139; 0.068	20.681; <0.001**	0.914; 0.822
Department			
Internal medicine	1.02±1.05	46.5±5.79	19.7±6.33
Surgery	1.40±1.01	45.9±5.90	19.9±6.47
General medicine	1.07±1.07	46.0±6.55	18.2±6.08
Other allied department	1.05±0.89	46.4±5.98	19.1±6.84
H-test; p-value^‡^	9.395; 0.024**	0.517; 0.915	3.638; 0.303
Do you have any interaction on a regular basis with someone who is Deaf besides the working environment?			
Yes	1.32±1.03	45.9±6.85	20.6±5.72
No	1.12±1.02	46.2±6.01	18.9±6.48
Z-test; p-value^§^	1.321; 0.186	0.141; 0.888	1.340; 0.180
Have you had any previous interactions with a Deaf patient?			
Yes	1.22±1.03	46.0±5.92	20.2±5.82
No	1.05±1.00	46.3±6.30	17.9±6.85
Z-test; p-value^§^	1.652; 0.099	0.475; 0.635	3.440; 0.001**
Do you have any type of sign language skills?			
Yes	1.13±1.01	45.4±6.37	20.9±6.52
No	1.14±1.02	46.3±6.04	18.8±6.35
Z-test; p-value^§^	0.026; 0.979	0.746; 0.456	2.299; 0.022**

**Figure 2:**
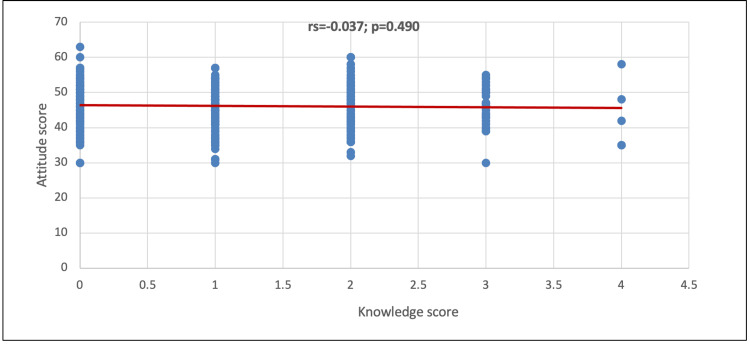
Correlation between the knowledge score and attitude score

In Figure 2, no significant correlation was observed between the knowledge score and attitude score (p=0.490).

**Figure 3:**
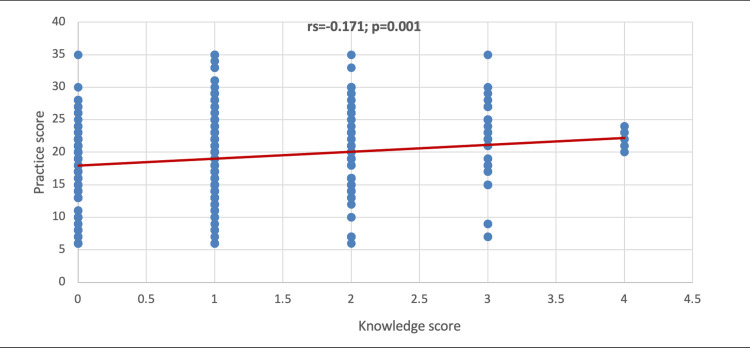
Correlation between the knowledge score and practice score

In Figure 3, it was shown that there was a positive significant correlation between the knowledge score and practice score (rs=0.171; p=0.001).

**Figure 4:**
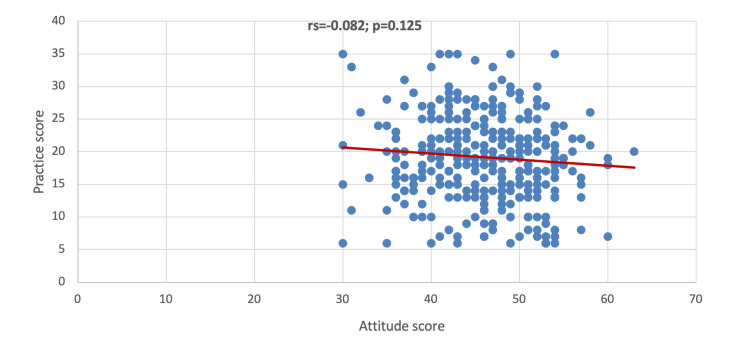
Correlation between the attitude score and practice score

In Figure 4, it was found that there was no significant correlation between attitude score and practice score (p=0.125).

## Discussion

The present study investigated HCWs' KAP toward Deaf adult patients. There were limited studies in Saudi Arabia that measured whether HCWs are equipped with adequate KAP or not when dealing with Deaf patients. Thus, the findings of this study would be of great addition to the literature. 

Level of knowledge

The knowledge of our participants regarding deafness was relatively low. Based on the given criteria, nearly 99% of our subjects were considered to have poor knowledge. None of our HCWs were deemed to have good knowledge levels (mean score: 1.14 out of 7 points). This is consistent with the study of Velonaki et al. [6], which indicated that nurses lacked knowledge about Deaf people, suggesting that only 5.8% were aware that Deaf patients were not conversant about their health issues. Supporting these reports, a qualitative report done by Orrie and Motsohi [13] revealed that HCWs had preconceptions and gaps in knowledge and awareness. They identified themes such as language barriers, improvisation and innovation, interpreters, resilience, and recommendations as the point of concern, adding that communication is a challenge, but HCWs claimed this barrier is not insurmountable.

Significant factor of knowledge

Data from our study suggest that HCWs working in the surgery department could be associated with better knowledge than those working in other departments. In spite of these results, the overall knowledge of all HCWs still has big gaps. In Puerto Rico [22], they reported that increasing knowledge was associated with increasing medical year level. However, among American medical students [21], comparing the knowledge before and after the educational workshop, it was found that medical students' knowledge increased significantly after the four-hour workshop. In addition, students were also shown to increase confidence in their interaction with the Deaf community. Thus, the author acknowledged that the confidence and/or short-term knowledge in the interaction with Deaf patients could be effectively improved by a single workshop.

Details of knowledge

Regarding the specific assessment of knowledge, our results highlight the huge gaps in HCWs' understanding of the basic facts of deafness, most notably about the number of signing Deaf people living in Saudi Arabia (correct answer: 4.6%) and the estimated prevalence of deafness in the Kingdom (correct answer: 3.1%). Surprisingly, none of our HCWs knew that genetic factors, maternal rubella, and meningitis were all common causes of deafness, and none of them were aware of the frequency of Deaf children having hearing parents. In Greece [6], 40.4% of the respondents knew that SL is international, and few of them were aware that it has its own syntax and grammar (12.2%). Incidentally, the use of a Deaf interpreter has been suggested by 19.2% of participants, while only 5.8% had the information that Deaf people may not be fully aware of their health issues. A previous literature published in the United States [12] documented that writing was the most prominent method of communication with Deaf patients. However, nearly two-thirds (63%) of physicians were aware that SL was primarily used during the initial screening of Deaf patients, while SL interpreters were not used more often than other methods in their practices.

Level of attitude

Incidentally, the level of attitude of our HCWs mirrored the results of the knowledge. Our data indicates that the majority had neutral attitudes (63.2%), but 36.8% were assumed to have negative attitudes. None of our HCWs were classified as having positive attitudes (mean score: 46.2 out of 90 points). This is comparable to the study of Devkota et al. [2] which found a low attitude of nurses toward disability and the experience of women with disabilities toward the utilization of maternal healthcare services. However, comparing the attitudes of medical students who underwent the SL module (case) and students who underwent different modules (control), it was revealed that after module completion, students who participated in the SL module had better attitudes and higher knowledge scores than the control group. 

Significant factor of attitude

Several demographic variables were seen to influence attitude, including nationality, marital status, having children, years of working experience, and specialty. This further indicates that being non-Saudi, being married, having children, increasing years of working experience, and nursing specialty were identified as the significant predictors of increased attitude. In Bhutan [3], study suggests that physicians were more associated with a better score on the SADP. However, no significant differences were observed between the attitude scores of male and female participants. In our study, the scores of attitude were not significantly different when compared to age and gender (p>0.05). Contradicting these reports, in the United Kingdom [19], there was a significant difference observed between the attitude of men and women (p<0.05), with women demonstrating a better attitude than men. 

Details of attitude

The lack of attitude of our respondents stemmed from the details of attitude. Based on a 5-point Likert scale response, the mean score was lower in the following statements (mean scores: <2 points out of 5 points): "Learning about communicating with deaf people is worthwhile," "Interpreters should be available for deaf people at hospitals," and "More research should be done to find cures for deafness." These statements are areas of most concern." In Chile [23], study suggests that health professionals who received some information about deafness exhibited a better attitude toward having more deaf friends, while in Malaysia [24], study reported that respondents had the attitude of writing back and forth to communicate with deaf patients which they thought is the most effective form of delivering information to deaf people. 

Level of practice

Despite shortcomings in both knowledge and attitude, HCWs' practices in dealing with Deaf patients were also deemed inadequate, with poor levels constituting 40.2%, while moderate levels were 45.9% and good levels were only 14%. To our knowledge, this is the first study in Saudi Arabia that managed to present HCWs' level of practice toward deaf patients. Hence, more investigations are warranted to validate these findings. 

Significant factor of practice

Significant factors of practice include male gender, previous interaction with deaf patients, and knowledge of any type of SL. However, we found no significant difference between the practice scores in relation to age, nationality, marital status, having children, years of working experience, specialty, and department (p>0.05). Incidentally, we noted a significant correlation between practice and knowledge scores (p=0.001). This further indicates that as knowledge increases, practice will also increase. However, we found no significant correlation between the score of practice and the score of attitude or the knowledge score and the attitude score (p>0.05). This is almost consistent with the study of Devkota et al. [2]. The difference in the mean score of those who participated in disability training and those who did not was not statistically significant (p>0.05); however, in a study by Cooper et al. [19], the knowledge of deafness did not seem to correlate with the attitudes toward deaf patients. Nevertheless, an association had been observed between professionals' interactions with deaf people of equal or higher status and more optimistic attitudes. 

Details of practice

When evaluating the details of HCWs' practices in dealing with Deaf patients, based on 5-point Likert scale responses, the lowest ratings were seen toward the items of "Able to understand some forms of Arabian SL" (mean score: 2.39) and "Able to effectively gather complaints of deaf patients" (mean score: 2.44). It is only in the item about "getting Deaf patients' attention" where our respondents demonstrated good ratings (mean score: 3.38 out of 5 points); other practice items were considered moderate to poor ratings. In Malaysia [24], about half of the participants have a misconception about the need for deaf patients' services, with a vast majority thinking that communication is a bit of a challenge when providing services among this population group. On the other hand, in Puerto Rico [22], the most common problem that deaf people might encounter during hospitalization is dealing with emergency events (i.e., fire alarms).

Previous interaction with Deaf patients

Previous interaction with Deaf people could be a contributing factor for KAP. In our study, more than half of our respondents had previous interactions with Deaf patients, while interactions with Deaf person outside of the working environment has been reported by 10.5%. In a qualitative report done by Orrie and Motsohi [13], HCWs had limited encounters with Deaf or hearing-impaired patients. Despite this scenario, HCWs greatly recommended SL training and courses along with the availability of deaf interpreter in their institution. 

SL skills

Another important contributing factor to KAP is having SL skills. In our study, only 15.4% had SL skills, with most of them using SL by hand (48.1%), which they learned online or through self-study (48.1%). Among Greek nurses [6], only 5.8% had ever attended a course related to disability, while attendance to SL courses was also deemed low (2.3%). This is contrary to the medical students in Puerto Rico [22], as 21% already attended the American Sign Language (ASL) course and 86% were interested in SL future-related courses.

## Conclusions

The KAP of HCWs toward Deaf patients was unsatisfactory. HCWs working in the surgery department were likely to demonstrate better knowledge than the other HCWs working in other departments. Nevertheless, the overall knowledge of all HCWs was suboptimal. Further, respondents who were married with children, had more years of experience, and were nurses in the profession were associated with better attitudes; however, male HCWs may exhibit better practices in dealing with Deaf patients than their female counterparts. This study provides evidence of the lack of SL education among our population. To address the gaps in KAP, we greatly recommend periodical staff training programs related to SL, which would be beneficial to both HCWs and Deaf patients. Hence, this study supports the recommendation of having Deaf interpreters at all times at the hospital facilities.

## Appendices

Online survey form of knowledge, attitude, and practice toward deaf patients among healthcare workers

Section 1: Consent Form

Dear Participant,

Thank you for accepting to take part in this online questionnaire survey. The purpose of this online survey is to estimate the level of knowledge, attitude, and practice toward deaf patients among healthcare workers. It will take approximately five to eight minutes to complete the survey. Be assured that all answers you provide will be kept in a strictest confidentiality. Please feel free to email Dr. Nurah Alamro and team at to answer your questions. If you are willing to participate this online survey, please click “Next” to begin.

Section 2: Demographics

1. What is your gender?

Female

Male

2. What is your age?  

18-27

28-37

38-47

48-57

>57

3. What is your nationality?

Drop menu

4. What is your current marital status?

Single

Married

Divorced

Widowed

5. Do you have any children? (relative)

Yes

No

6. If you answered yes to the previous question, does anyone of your children have any disabilities?

Yes

No

7. If you answered yes, please determine the type of disability:

8. Beside the working environment, do you have any interaction on a regular basis with someone who is deaf?

Yes

No

9. If you answered yes, please specify whether this interaction was with a:

Family member

Friend

Community

Others (please specify):

10. What is your occupation?

Dentist

Intern

Laboratory worker

Nurse 

Nutritionist

Pharmacist

Physician

Physiotherapist

Technician

Others (please specify):

11. What is your department? 

Anesthesia

Critical Care

Dermatology

Ear, Nose, and Throat (ENT)

Emergency Medicine

Family and Community Medicine

Internal Medicine

Obstetrics and Gynecology

Ophthalmology

Pediatrics

Psychiatry

Radiology

Surgery

Others (please specify):

12. How many years of experience do you have?

<1 year

1-5 years

6-10 years

>10 years

13. Have you had any previous interaction with a deaf patient?

Yes

No

14. Do you have any sign language skills? 

Yes

No

15. If you answered yes to the previous question, what type of sign language do you possess? (text box) 

16. From where did you learn your sign language skills? 

Family/friends

College/community course

Online/self-study

Others (please specify):

17. What level of Saudi Sign Language skills do you possess? 

None

Minimal

Basic

Intermediate 

Advanced 

Section 3: Knowledge

The following questions are designed to ascertain your knowledge on deafness. 

18. What is the language most used by people who are deaf in Saudi Arabia?

Universal Sign Language

Saudi Sign Language

Arabic Sign Language

None of the above

I don't know

19. What is the approximate prevalence of deaf and hard of hearing in Saudi Arabia?

1%

5%

9%

13%

I don't know 

20. Approximately how many deaf people are there in Saudi Arabia?

30,000

60,000

90,000

120,000

I don't know 

21. Which of the following is a common cause of deafness?

Genetic factors   

Maternal rubella

Meningitis

All of the above

I don't know 

22. Which of the following are deaf people NOT allowed to do in Saudi Arabia?

Join the police force

Drive a car

Be a witness in court

None of the above 

I don't know 

23. What is the unit that measures deafness?

Decibel

Frequencies

% of loss

Ampere

I don't know 

24. What is the percentage of deaf children who have hearing parents? 

30%

50%

70%

90%

I don't know 

*Section 4: Attitude* 

The following questions aim to assess different domains of attitudes toward deafness. Please take your time considering your most suitable choices.

25. I have no confidence in communicating with deaf people.

Strongly agree

Agree

Neutral

Disagree

Strongly disagree

26. I would like to communicate with deaf people.

Strongly agree

Agree

Neutral

Disagree

Strongly disagree

27. I would like to take a sign language course to communicate with deaf people. 

Strongly agree

Agree

Neutral

Disagree

Strongly disagree

28. Communicating with deaf people would make me very nervous.

Strongly agree

Agree

Neutral

Disagree

Strongly disagree

29. I think communicating with any deaf would be enjoyable and stimulating.

Strongly agree

Agree

Neutral

Disagree

Strongly disagree

30. Learning about communicating with deaf people is worthwhile. 

Strongly agree

Agree

Neutral

Disagree

Strongly disagree

31. Communicating with deaf people makes me feel uncomfortable.

Strongly agree

Agree

Neutral

Disagree

Strongly disagree

32. I am sure I could learn to communicate with deaf people effectively.

Strongly agree

Agree

Neutral

Disagree

Strongly disagree

33. I think communicating with deaf people would be very hard for me. 

Strongly agree

Agree

Neutral

Disagree

Strongly disagree

34. Deaf people should learn to lipread.

Strongly agree

Agree

Neutral

Disagree

Strongly disagree

35. Deaf people should learn speech rather than sign language.

Strongly agree

Agree

Neutral

Disagree

Strongly disagree

36. Once I started to communicate with deaf people, I would probably want to have more opportunities to use it. 

Strongly agree

Agree

Neutral

Disagree

Strongly disagree

37. Knowing how to communicate with deaf people will increase my opportunities. 

Strongly agree

Agree

Neutral

Disagree

Strongly disagree

38. It is important to me to communicate with deaf people. 

Strongly agree

Agree

Neutral

Disagree

Strongly disagree

39. Interpreters should be available for deaf people at hospitals.

Strongly agree

Agree

Neutral

Disagree

Strongly disagree

40. Deaf people should receive help in their home environment.

Strongly agree

Agree

Neutral

Disagree

Strongly disagree

41. Deaf people should not be viewed as "impaired." 

Strongly agree

Agree

Neutral

Disagree

Strongly disagree

42. More research should be done to find cures for deafness.

Strongly agree

Agree

Neutral

Disagree

Strongly disagree

*Section 5: Practice* 

43. I am able to effectively get the deaf patients' attention (tapped on shoulder, waved, etc.).

Always 

Usually 

Sometimes 

Rarely

Never

44. I am able to introduce myself to deaf patients.

Always 

Usually 

Sometimes 

Rarely

Never

45. I am able to effectively gather the deaf patients' complaint (feels sick, head hurts, etc.).

Always 

Usually

Sometimes 

Rarely

Never

Not applicable 

46. I am able to successfully recognize and understand the following Saudi Sign Language signs: pain, sick, and help.

Always 

Usually 

Sometimes 

Rarely

Never

47. I am able to ask deaf patients to wait for an interpreter.

Always 

Usually 

Sometimes 

Rarely

Never

48. I am able to check for deaf patients' understanding.

Always 

Usually 

Sometimes 

Rarely

Never

49. I am able to refrain from "offensive" actions or behaviors such as exaggerating speech, interrupting, and talking over deaf patients.

Always 

Usually 

Sometimes 

Rarely

Never

50. What is the most common method that you use during your communication with deaf patients? 

Interpreter

Lipreading

Writing back and forth

Through a patient's relative or friend as an interpreter

Sign language

Gesture between physician and patient

Others (please specify):

Section 6: Closure

Thank you for your participation.

Optional: Provide your email address if you want to get the research results.
